# Effectiveness of perioperative oral hygiene management using a cetylpyridinium chloride‐, dipotassium glycyrrhizinate, and tranexamic acid‐based mouthwash as an adjunct to mechanical oral hygiene in patients with maxillomandibular fixation: A randomized controlled clinical trial

**DOI:** 10.1002/cre2.814

**Published:** 2023-11-28

**Authors:** Reona Aijima, Yoshio Yamashita

**Affiliations:** ^1^ Department of Oral and Maxillofacial Surgery, Faculty of Medicine Saga University Saga Japan

**Keywords:** cetylpyridinium chloride, dipotassium glycyrrhizinate, mouthwash, tranexamic acid

## Abstract

**Objectives:**

Maxillomandibular fixation requires the jawbones to remain static. Mechanical cleaning is also carried out by brushing or with a water flosser to maintain the oral cavity in a hygienic state, but this cannot be considered sufficient. Mouthwashes are used as a substitute for mechanical cleaning or in a supplementary role after such cleaning. The aim is to evaluate the effectiveness of HABITPRO mouthwash, which contains cetylpyridinium chloride, dipotassium glycyrrhizinate, and tranexamic acid in the specific environment created by maxillomandibular fixation used as an adjunct to mechanical cleaning.

**Material and Methods:**

A total of 55 patients who had undergone maxillomandibular fixation were randomly allocated to either a HABITPRO group (*n* = 29) or a placebo group (*n* = 26). To investigate their oral hygiene status, their plaque control record (PCR) was reviewed, and the caries‐related bacterial counts, pH, acid buffering capacity, white blood cell count, and ammonia in saliva were measured immediately before maxillomandibular fixation, on Day 10 of fixation, and immediately after fixation was released.

**Results:**

After approximately 2–3 weeks of mouthwash use, the PCR index also increased significantly in the placebo group compared with baseline, whereas it remained almost steady in the HABITPRO group. Additionally, salivary ammonia levels decreased significantly in the HABITPRO group compared to that of the placebo group.

**Conclusions:**

Even with maxillomandibular fixation, continued gargling with HABITPRO mouthwash in the perioperative period as an adjunct to mechanical cleaning can help maintain better oral hygiene and reduce bacterial counts.

## 1 INTRODUCTION

It has long been known that the oral cavity is a breeding ground for bacteria, because it is not only maintained at a constant temperature, but it is also a moist environment (Aas et al., 2005). The intraoral microbiota is known to change in accordance with the intraoral environment (Akiyama et al., 2010; Sano et al., 2012; Takeshita et al., 2015). As is well known, this means that the environment in the mouth, which anatomically is the entrance to both the digestive tract and the respiratory system, has a range of systemic effects. In addition to the diseases of aspiration pneumonia and diabetes mellitus, its contribution to frailty has also come under scrutiny in recent years (Lu & Yang, 2004; Tanaka et al., 2018; Xue et al., 2008; Yoneyama et al., 1999).

Under normal circumstances when homeostasis is maintained, individuals are able to coexist with the commensal resident intraoral biota by means of their own immunity and natural purification. However, diminished immunity in the periprocedural period (whether the procedure involves surgery, radiotherapy, chemotherapy, or other treatment) or due to age means that homeostasis can no longer be maintained, and a range of problems can occur, including wound infection, stomatitis and resulting secondary infection, caries, and periodontitis. Stomatitis is a particular issue during radiotherapy and chemotherapy, and since it causes bleeding and pain, mechanical cleaning becomes difficult (Saadeh, 2005). In addition, if food consumption by mouth decreases as a result of mucosal inflammation, nutritional status worsens, and the healing of this mucosal inflammation is delayed in a vicious circle.

Special conditions caused by surgery are also known to affect the oral environment in various ways. For example, patients with a mandibular fracture and those who have undergone orthognathic surgery may undergo maxillomandibular fixation to encourage bony healing, but this means that they are unable to open their mouths and cannot eat by mouth for a certain period. The most common complication after these surgeries is postoperative infection. Antimicrobial administration plays an important role in the prevention of postoperative infections, but carries the risk of diarrhea and the increasing bacterial resistance (Thornhill et al., 2015). Thus, reducing of oral bacterial counts is of paramount importance.　During this fixation period, physical oral cleaning is inadequate, and the oral environment deteriorates. Brushing and water flossing are used for mechanical cleaning, but this cannot be considered sufficient. Under these conditions, chemical cleaning by gargling with mouthwash has long been used as a supplementary cleaning method. There are several comparative studies of postoperative infection with antimicrobials in intermaxillary fixation patients, but there are no comparative studies with mouthwash (Davis et al., 2017; Schaller et al., 2013).

HABITPRO mouthwash contains the following three active ingredients: cetylpyridinium chloride (CPC), dipotassium glycyrrhizinate (GK2), and tranexamic acid (TXA), which are expected to have bactericidal, anti‐inflammatory, and hemostatic effects.

The objective of this study was to investigate the effect of continued perioperative use of HABITPRO mouthwash with mechanical cleaning on the intraoral environment, particularly in patients undergoing maxillomandibular fixation, which is a specific treatment used in dental and oral surgery. Specifically, the intraoral environment was evaluated by testing the amount of dental plaque and salivary properties to investigate the effectiveness of this mouthwash during maxillomandibular fixation.

## 2 PATIENTS AND METHODS

In this study, single‐blind participants are blinded to the parallel group that was initiated within a single institution. The study was approved by the Saga University Hospital Research Ethics Committee (Protocol number 2019‐07‐01), and informed consent was obtained from all subjects and/or legally acceptable representative (LAR) of subjects to participate in the study.　This trial was registered at the University Hospital Medical Information Network (UMIN) Clinical Trials Registry (Registration number UMIN000046091). This study followed the 2010 Consolidated Standards for Reporting Trials (CONSORT) statement.　The study was performed in line with the principles of the declaration of Helsinki. Regarding setting sample size, there were few similar research reports in the past. This study was conducted under the actual conditions of daily clinical practice, and the sample size was set as the number of possible cases within the study period.

### 2.1 Participants and study design

The study subjects were patients in the Department of Dental and Oral Surgery of Saga University Hospital (Saga, Japan) who required maxillomandibular fixation either after orthognathic surgery or for the treatment of mandibular fracture between October 2019 and December 2022. The subjects were allocated by the envelope method to either the HABITPRO group or the placebo group. Patients with severe undernutrition (Alb ≤2.5 g/dL) or those who were unable to gargle unaided were excluded from the study (Table 1). During maxillomandibular fixation, they received transnasal enteral nutrition, with only water taken by mouth. The duration of fixation was 14–20 days. The HABITPRO group gargled with a mouthwash containing active ingredients (CPC, GK2, and TXA), and the placebo group with a mouthwash that did not contain any active ingredients (both mouthwash were provided by Earth Corporation). Both types of mouthwash were formulated so that there was no difference between them in factors such as color and taste. The procedure for intraoral cleaning started with mechanical cleaning with manual toothbrush using benzethonium chloride and a water flosser (Waterpik, Water Pik Inc.) for approximately 5 min, followed by gargling with the allocated mouthwash. No interdental cleaning aids were used. The cleaning was conducted four times a day after enteral nutrition (7:00, 11:00, 15:00, and 19:00), and the mouthwash was held and rinsed in the mouth for 30 s before it was spat out. A mondamin automatic dispenser (Earth Corporation) was used to dispense 20 mL for each use.

**Table 1:** Exclusion criteria.

(1) Having severe undernutrition from before the start of treatment (Alb ≤2.5 g/dL)
(2) Unable to gargle independently
(3) Considered by an investigator to be unsuitable to participate in the study for any other reasons

### 2.2 Evaluation parameters

(1) Assessment of plaque adhesion status using the plaque control record (PCR).
(2) Measurements with a saliva analyzer (Sill‐Ha, Arkray Marketing Inc.).

Plaque‐disclosing dye (Shofu Inc.) was used to dye all the remaining tooth surfaces, and the state of plaque adhesion on the surface in the cervical region was scored. The method of implementation was prescribed for four tooth surfaces (buccal, mesial, distal, and lingual/palatal) before and after intermaxillary fixation, and for three tooth surfaces (buccal, mesial, and distal) during intermaxillary fixation, and multiple clinicians were assigned without blinding. Records were recorded on a standardized paper chart, and the analysis was calculated as plaque‐positive tooth surface/total tooth surface × 100 and was evaluated by one person in a blinded fashion. The saliva analyzer uses patients' saliva to measure six parameters related to caries (carious bacteria, pH, and acid buffering capacity), periodontal disease (leukocyte count and protein levels), and oral cleanliness (ammonia levels). Specifically, saliva was collected after the mouth had been washed out with 3 mL of water for 10 s. Drops of saliva were placed on a special test paper, which was measured by the analyzer. The ammonia level was measured by the glutamate dehydrogenase assay. Measurements (1) and (2) were made at three time points: immediately before maxillomandibular fixation (baseline); on Day 10 of fixation; and immediately after fixation was released. There was individual variation between patients in the intraoral environment at baseline. Therefore, the amount of change in each measured value over time was calculated and compared. In this study, the primary outcome of this study was focused on PCR to evaluate oral hygiene status. Along with the primary outcome, six parameters related to caries, periodontal disease, and oral cleanliness in saliva were assessed.

### 2.3 Statistical analysis

JMP version 14 was used for statistical analysis. The primary and secondary endpoints were compared between the HABITPRO and placebo groups. Student's *t*‐test was used for continuous variables, and Fisher's exact test for nominal scales. The *p*‐value was set to the level of significance of 0.05.

## 3 RESULTS

### 3.1 Study flow and baseline characteristics

**Figure 1:**
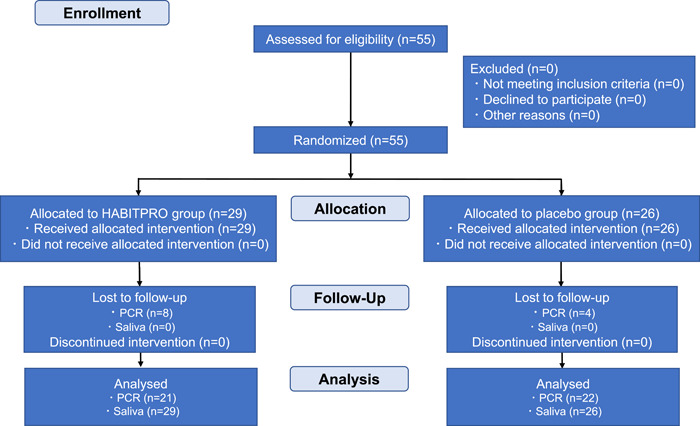
Flow diagram of patients throughout the study.

The flow of patients throughout this study is depicted in Figure 1. A total of 55 patients who had undergone maxillomandibular fixation were randomly allocated to either a HABITPRO group (*n* = 29) or a placebo group (*n* = 26). No side effects were observed in any group during the intervention. The study subjects were patients who required maxillomandibular fixation either after orthognathic surgery (*n* = 44) or for the treatment of mandibular fracture (*n* = 11). They included 24 men and 31 women, aged 16–81 years (mean age 28.11 years). PCR data were analyzed for 43 of the 55 patients from whom these data could be collected due to difficulty during maxillomandibular fixation (22 patients in the placebo group and 21 in the HABITPRO group). There was no difference between the two groups in sex, smoking habits, age, disease, or number of teeth (Table 2). There were no reports of irritation‐caused pain or signs of mucosal inflammation in either the HABITPRO group or the placebo group. No patient dropped out of the study early.

**Table 2:** Patient distribution.

	Placebo	HABITPRO	*p*‐Value
Gender			
Male/female	12/14	12/17	0.789
Smoking habits			
Yes/no	3/23	4/25	1.000
Age	28.23 (13.52)	28.00 (15.78)	0.954
Disease			
Jaw deformity/fracture	21/5	23/6	1.000
Number of teeth	26.38 (1.92)	25.72 (3.22)	0.366

*Note*: Data represent the mean ± (SD).

### 3.2 The effectiveness of HABITPRO mouthwash on plaque control record

**Figure 2:**
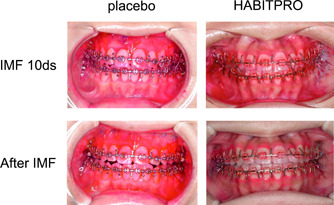
Representative intraoral photograph on Day 10 of fixation and after fixation release. Plaque‐disclosing dye was used to dye all the tooth surfaces.

To assess the intraoral environment, the amount of dental plaque was evaluated using PCR as the primary endpoint. The PCR index varied widely at baseline. After fixation release, in the placebo group, the index increased by +24.61 compared with baseline, with an increase in the amount of plaque adhering to the teeth, whereas in the HABITPRO group, it was almost unchanged from baseline after fixation release, decreasing by –2.00 (Table 3). The difference between the two groups was significant　(Figure 2).

**Table 3:** Plaque control record.

	Placebo *n* = 22 mean (SD)	HABITPRO *n* = 21 mean (SD)	*p*‐Value
Pre	42.18 (17.26)	57.77 (26.15)	0.026*
IMF 10ds	79.26 (24.05)	83.17 (20.78)	0.573
After IMF	66.79 (31.41)	55.77 (26.65)	0.223
Δ (Pre‐IMF 10ds)	−37.08 (31.27)	−25.40 (24.18)	0.179
Δ (Pre‐after IMF)	−24.61 (35.53)	2.00 (27.71)	0.009*

Abbreviation: IMF, intermaxillary fixation.

* *p* < 0.05.

### 3.3 The effectiveness of HABITPRO mouthwash on salivary properties

We investigated six parameters in saliva (carious bacteria, pH, acid buffering capacity, leukocyte count, protein levels, and ammonia levels) to verify whether HABITPRO mouthwash affects caries, periodontal disease, and oral cleanliness (Table 4). Interestingly, in the placebo group, the ammonia level related to oral cleanliness after fixation release increased by +5.42 compared with baseline. In the HABITPRO group, a marked decrease of –14.66 was evident. This difference between the two groups was significant. This result was consistent with the finding that the PCR index in the HABITPRO group remained stable and did not worsen after intermaxillary fixation was released.

**Table 4:** Salivary properties.

	Placebo *n* = 22 mean (SD)	HABITPRO *n* = 21 mean (SD)	*p*‐Value
*S. mutans* metabolic activity			
Δ (Pre‐IMF 10ds)	−11.85 (32.93)	0.55 (26.36)	0.127
Δ (Pre‐after IMF)	−9.85 (33.45)	3.62 (32.76)	0.138
Acidity			
Δ (Pre‐IMF 10ds)	5.69 (30.73)	8.00 (34.72)	0.796
Δ (Pre‐after IMF)	4.96 (27.15)	2.03 (28.79)	0.701
Buffer capacity			
Δ (Pre‐IMF 10ds)	−8.69 (34.20)	3.14 (32.04)	0.191
Δ (Pre‐after IMF)	−4.11 (31.47)	10.07 (27.81)	0.082
Leukocyte levels			
Δ (Pre‐IMF 10ds)	−8.84 (36.19)	−3.28 (27.84)	0.523
Δ (Pre‐after IMF)	−4.39 (31.61)	−0.03 (36.21)	0.666
Protein levels			
Δ (Pre‐IMF 10ds)	3.85 (26.05)	−8.00 (29.29)	0.121
Δ (Pre‐after IMF)	4.27 (28.75)	−6.07 (26.12)	0.168
Ammonia levels			
Δ (Pre‐IMF 10ds)	−13.00 (22.41)	−3.76 (32.14)	0.227
Δ (Pre‐after IMF)	−5.42 (21.21)	14.66 (34.93)	0.014*

Abbreviation: IMF, intermaxillary fixation.

* *p* < 0.05.

The buffering capacity of saliva increased similarly by +4.11 compared with baseline in the placebo group, but decreased by –10.07 in the HABITPRO group. However, this difference between the two groups was not significant. There were no significant differences between the two groups in pH, carious bacteria, leukocyte count, or protein levels at any time point.

## 4 DISCUSSION

Recent years have seen rising interest in oral health management. Although the rate of mouthwash use is not necessarily higher in Japan than in other countries, it has tended to increase in recent years. Initially, most people thought that mouthwash was used to eliminate bad breath, and it was often lumped together with fragrances, so that rather than using it regularly, people tended to use it occasionally in a way that was greatly removed from the goal of health management. Recently, however, it has gradually come to be thought of differently, as awareness has spread of the necessity of oral function management. The main uses of mouthwash now include cleaning the mouth, retaining moisture, preventing caries and periodontal disease, and preventing bad breath.

Since oral intake is not performed during maxillomandibular fixation, it is known that saliva secretion decreases and oral dryness becomes more severe. Additionally, it has been reported that oral bacterial flora changes during tube feeding, leading to an increase in aspiration pneumonia (Finucane & Bynum, 1996). In addition, oral hygiene becomes even more difficult during intermaxillary fixation. Mechanical cleaning with brushing and water flossing is important, but this can not be sufficient. During intermaxillary fixation, chemical cleaning using mouthwash is thought to be effective, but there are no reports about this matter. In our study, the PCR index significantly increased during intermaxillary fixation compared to that before surgery, suggesting that oral hygiene worsened.

The mouthwash investigated in this study is compounded with three medicinal ingredients. As a cationic surfactant, CPC exerts a bactericidal action by adsorption on the bacterial cell membrane, which increases its fluidity and causes it to break down (Hwang et al., 2013; Ioannou et al., 2007). It is also believed to have a bactericidal effect by denaturing proteins. GK2 is a natural ingredient derived from licorice recognized for its anti‐inflammatory properties, that is used to suppress stomatitis and throat inflammation (Michaelis et al., 2011). Additionally, it has been reported that GK2 inhibits inflammation and ameliorates colitis in mice (Vitali et al., 2013). TXA is an artificial synthetic amino acid that is expected to have hemostatic and anti‐inflammatory actions (Jimenez et al., 2007; Kämmerer et al., 2015). Recently, it has been reported that TXA mouthwash is effective for preventing postoperative bleeding after tooth extraction (Carter et al., 2003). Previously, efficacy of CPC‐based mouthwash and CPC/TXA‐based mouth rinse have been reported (Costa et al., 2013; Lee et al., 2017). Depending on the proportions in which these medications are combined, their pharmacological effects can cancel each other out. This CPC/GK2/TXA‐based mouthwash has been confirmed by the manufacturer's development department to ensure that the ingredients do not cancel each other out. Additionally, CPC/GK2/TXA‐based mouthwash inhibited propagation of the bacteria extracted from the postsurgical sutures after implant placement (Taninokuchi et al., 2021).

In the present study, the effectiveness of this mouthwash was assessed in a specific environment resulting from maxillomandibular fixation. The results of the PCR experiments suggested that the use of a mouthwash containing active ingredients may suppress the adhesion of debris in comparison with the placebo group. During maxillomandibular fixation, all of the patients in this study carried out mechanical flossing with a Waterpik before using mouthwash. In both groups, the labial‐side tooth surfaces and mucosa were thus adequately cleaned by mechanical stimulation, but the present results showed that this alone was insufficient. The ammonia levels and buffering capacity of saliva were also affected by the mouthwash ingredients, despite Waterpik use. This may have been because the action of the Waterpik did not extend to the lingual‐side tooth surfaces or the tongue and other parts of the mucosa. A previous study found that the use of this mouthwash reduced debris adhesion to the sutures used in implant surgery, which supports the present results (Taninokuchi et al., 2021). Salivary ammonia is thought to be generated by the metabolism of urea or amino acids by the ureases and amino acid‐metabolizing enzymes of oral bacteria (Kanapka & Kleinberg, 1983; Shu et al., 2007; Singer & Kleinberg, 1983). Salivary ammonia concentration is associated with the total oral bacterial count, and gram‐negative bacilli produce particularly large amounts of ammonia. Gargling with this mouthwash effectively reduced the amount of ammonia in saliva, and it may significantly suppress these bacteria. The study subjects were generally young, with a mean age of 28 years, but the ammonia concentration in saliva has been shown to increase in accordance with age from age 40 years, which suggests that a greater effect might be obtained in older people. However, this is a topic for further study.

Unlike oral medications, including antibiotics, mouthwash exerts direct local action to reduce systemic side effects. Therefore, mouthwash can be used continuously for a wide range of indications and used by a wide range of age patients. Barriers to the use of mouthwashes include irritation of the mucous membranes and their smell and taste. Therefore, taste and color can be important factors when choosing a mouthwash. In this study, the drug was used in patients ranging in age from 10 to 80 years and there were no adverse events such as mucositis or pain. In addition, there were no patients who were unable to use the product due to taste or odor preferences.

The importance of oral management in the perioperative period is now recognized in both medicine and dentistry, and active interventions are now being performed (Bergan et al., 2014; Soutome et al., 2017). However, what must not be forgotten is that gargling is the basis of oral hygiene management, and moreover that it is essential that this become a daily habit, so that patient education is important. Symptoms such as a refreshing sensation in the mouth and demonstrating the results of oral hygiene management to patients who use mouthwash can be a factor in motivating and encouraging them to continue. Mouthwash use can also be effective when normal oral cleaning is difficult, such as in times of disaster or in long‐term care settings. It is generally marketed to prevent bad breath and is widely available for this purpose, but the type of mouthwash to be used must be chosen in light of its intended use. The mouthwash in the present study played a supplementary role in maintaining the oral environment after mechanical cleaning, and further improvements to its ingredients and composition are required. Of course, some active ingredients will give rise to cases of hypersensitivity. A certain percentage of adverse events is also known to occur as a result of povidone‐iodine, benzethonium chloride, and other active ingredients that have been widely used.

During long‐term use, cost is another issue that must be resolved alongside taste preferences and irritation. The development of a versatile mouthwash for use not just in the perioperative period will be very important in terms of meeting a social need.

## 5 CONCLUSIONS

The present results suggest that the continued perioperative use of a mouthwash containing active ingredients may suppress the deterioration of the oral environment to some extent, even in the specific environment resulting from maxillomandibular fixation. This mouthwash may therefore provide a tool for perioperative oral hygiene management.

## 6 CLINICAL RELEVANCE

### 6.1 Scientific rationale for the study

Management of the oral environment in patients with diseases of oral and maxillofacial region is very important to reduce not only the aggravation of dental caries and periodontal disease, but also local wound infections and aspiration pneumonia.　Patients who required maxillomandibular fixation for the treatment of mandibular fracture or orthognathic surgery, a stronger oral environment management is required. Mouthwashes are used as a substitute for mechanical cleaning or in a supplementary role after such cleaning. fixation.　However, little is known about the effectiveness of mouthwash in the specific environment created by maxillomandibular fixation.

### 6.2 Principal findings

CPC/GK2/TXA‐based mouthwash reduced the ammonia level related to oral cleanliness in saliva and the PCR index after fixation release.

### 6.3 Practical implications

The continued use of CPC/GK2/TXA‐based mouthwash may suppress the deterioration of the oral environment, even in the specific environment resulting from maxillomandibular fixation. This mouthwash may be useful in perioperative oral hygiene management.

## AUTHOR CONTRIBUTIONS

Yoshio Yamashita designed the study, Reona Aijima performed the experiments, all authors interpreted data, and Yoshio Yamashita wrote the manuscript. All authors reviewed the manuscript.

## CONFLICT OF INTEREST STATEMENT

The authors declare no conflict of interest.

## Data Availability

The data that support the findings of this study are available from the corresponding author upon reasonable request.
